# Novel Thermostabilizing Formulation for Enhanced Stability of Foot-and-Mouth Disease Virus During Storage and Environmental Stress

**DOI:** 10.3390/microorganisms14092051

**Published:** 2026-09-14

**Authors:** Mi-Kyeong Ko, Yoon-Hee Lee, Su-Mi Kim, I-Ryeong Yang, Tae-jun Kim, Sung-Han Park, Jong-Hyeon Park, Saekil Yun, Seokin Yoon, Sung-Sik Yoo, Ji-Hyeon Hwang, Choi-Kyu Park

**Affiliations:** 1 Center for Foot and Mouth Disease Vaccine Research, Animal and Plant Quarantine Agency, 177 Hyeoksin 8-ro, Gimcheon 39660, Republic of Korea; mkk80@korea.kr (M.-K.K.); lyhee74@korea.kr (Y.-H.L.); iryoung0118@korea.kr (I.-R.Y.); taejun0526@korea.kr (T.-j.K.); shpark1124@korea.kr (S.-H.P.); parkjhvet@korea.kr (J.-H.P.); 2 Foot-and-Mouth Disease Diagnostic Division, Animal and Plant Quarantine Agency, 177 Hyeoksin 8-ro, Gimcheon 39660, Republic of Korea; beliefsk@korea.kr; 3 Choong Ang Vaccine Laboratories Co., Ltd. (CAVAC), Daejeon 34055, Republic of Korea; sk.yun@cavac.co.kr (S.Y.); siyoon@cavac.co.kr (S.Y.); saintyoo@cavac.co.kr (S.-S.Y.); 4 College of Veterinary Medicine & Animal Disease Intervention Center, Kyungpook National University, Daegu 41566, Republic of Korea

**Keywords:** foot-and-mouth disease virus (FMDV), stabilizer, thermostability, master vaccine seed

## Abstract

Among the seven-foot-and-mouth disease virus (FMDV) serotypes, serotype O is relatively thermolabile, posing significant challenges for virus preservation and vaccine manufacturing. Therefore, this study aimed to develop an effective thermostabilizing formulation to enhance the thermal stability of thermolabile FMDV serotype O strains, thereby facilitating their long-term storage. The stabilizing effect of a newly developed stabilizer, designated CVS, was assessed using two Korean field isolates (O/SKR/Jincheon/2014 and O/SKR/Boeun/2017) and two recombinant strains [O1 Manisa (R) and O PanAsia-2 (R)]. CVS was compared with conventional stabilizers consisting of 3% sucrose and 10% glycerol. Viral seed stocks were stored at −80 °C, 4 °C, 25 °C and 37 °C for 2, 4, and 7 d or subjected to accelerated stress conditions, after which viral thermostability was comparatively evaluated. Although 3% sucrose provided moderate protection, the CVS formulation demonstrated significantly superior stabilizing efficacy on viral infectivity, particularly under high-temperature stress conditions (25 °C and 37 °C) and during freeze–thaw cycles. However, the stabilizing effect varied among strains, suggesting strain-dependent differences in physicochemical properties and structural stability. Overall, CVS improved FMDV infectivity stability across a broad temperature range and exhibited greater protective efficacy than 3% sucrose, supporting its potential as an effective FMDV stabilizer.

## 1. Introduction

Foot-and-mouth disease (FMD) is one of the most economically devastating transboundary animal diseases affecting cloven-hoofed livestock, including cattle, pigs, sheep, and goats [1,2]. It is caused by foot-and-mouth disease virus (FMDV), a positive-sense, single-stranded RNA virus belonging to the genus *Aphthovirus* within the family *Picornaviridae* [3,4]. Vaccination remains the most effective strategy for preventing and controlling FMD, particularly in endemic countries where routine vaccination programs are implemented [5,6,7].

Since the first outbreaks of FMD in the Republic of Korea (ROK) in 2000, sporadic epidemics have continued through 2026. Following the devastating nationwide outbreak in 2010–2011, which resulted in substantial economic losses, compulsory vaccination of all cloven-hoofed animals was introduced in December 2010 [8]. The national FMD vaccination program currently depends entirely on imported vaccines, with annual expenditures exceeding USD 53 million [9,10]. Consequently, in the ROK, efforts are underway to establish domestic FMD vaccine production infrastructure and develop indigenous vaccine strains and manufacturing technologies. In particular, securing and maintaining a reliable master seed virus is an important prerequisite for the domestic production of FMD vaccines. To this end, vaccine candidate strains have been continuously developed and maintained from FMDV isolates obtained from both domestic outbreaks and international sources [11,12].

Commercial inactivated FMD vaccines are produced through the large-scale propagation of cell culture-adapted FMDV strains, followed by chemical inactivation while preserving the structural integrity of the viral capsid [13,14]. However, FMDV is inherently thermolabile and highly susceptible to environmental stresses, including elevated temperatures, pH fluctuations, and repeated freeze–thaw cycles [15]. Consequently, viral capsid degradation can significantly compromise vaccine efficacy and decrease antigen recovery during manufacturing [16,17]. To enhance viral stability, various stabilizing agents, including sugars, polyols, amino acids, proteins, and synthetic polymers, have been incorporated into preservation formulations [18,19]. Among these, sucrose and glycerol are widely used because of their cryoprotective and osmoprotective properties during vaccine production and storage [20]. However, these conventional stabilizers provide only limited protection against thermal stress, particularly at ambient (25 °C) and physiological (37 °C) temperatures and following repeated freeze–thaw cycles [21,22]. Moreover, some stabilizing compounds may adversely affect host cell viability, interfere with viral replication, or require additional downstream purification processes, thereby increasing manufacturing complexity and production costs [23,24]. Accordingly, novel stabilizing formulations capable of preserving FMDV infectivity and structural integrity under diverse environmental conditions while remaining fully compatible with cell culture-based vaccine production systems are required [25].

In the present study, we evaluated a novel thermostabilizing formulation, designated CVS, designed to enhance the stability of cell-adapted FMDV through optimization of its ionic composition, buffering capacity, and protective excipients. The thermostabilizing efficacy of CVS was evaluated using two FMDV serotype O field isolates from the ROK (O/SKR/Jincheon/2014 and O/SKR/Boeun/2017) and two recombinant vaccine strains [O1 Manisa (R) and O PanAsia-2 (R)]. The performance of CVS was systematically compared with that of conventional stabilizing formulations under various storage temperatures to assess its capacity to improve viral stability. Our findings demonstrate that CVS effectively preserves FMDV infectivity under various environmental stresses while exhibiting excellent biocompatibility with cell culture systems, supporting its potential application in FMD vaccine production and storage.

## 2. Materials and Methods

### 2.1. Cells

ZZ-R 127 cells (CCLV-RIE 0127, RRID: CVCL_5418), a fetal goat tongue epithelial cell line obtained from the Friedrich-Loeffler-Institut, Germany, were maintained in Dulbecco’s Modified Eagle’s Medium/F12 (Corning, Tewksbury, MA, USA; Cat. No. 10-090-CV, whereas baby hamster kidney (BHK-21) (C-13) adherent cells (Clone 13, ATCC CCL-10; ATCC, Manassas, VA, USA) and porcine kidney (LF-BK) cells obtained from the Plum Island Animal Disease Center (New York, NY, USA), were maintained in Dulbecco’s Modified Eagle Medium (Corning, Manassas, VA, USA; Cat. No. 10-013-CV). All cells were cultured in medium supplemented with 10% fetal bovine serum (ATLAS Biologicals, Fort Collins, CO, USA; Cat. No. FP-0500-A) and 1% antibiotic–antimycotic (Gibco, Paisley, UK; Cat. No. 15240-062) at 37 °C in an incubator with 5% CO_2_ atmosphere. BHK-21 suspension cell co-developed by the Animal and Plant Quarantine Agency and the Korea Research Institute of Bioscience and Biotechnology was cultured in either ProVero-1 (Lonza, Basel, Switzerland; Cat. No. BEBP02-030Q) or CD BHK-21 production medium (Gibco, Thermo Fisher Scientific, Waltham, MA, USA; Cat. No. A16277-01). ProVero-1 medium was supplemented with dextran sulfate (Sigma-Aldrich, St. Louis, MO, USA; Cat. No. D8906), L-glutamine (Thermo Fisher Scientific, Waltham, MA, USA; Cat. No. A29168-01), Pluronic F-68 (Thermo Fisher Scientific, Waltham, MA, USA; Cat. No. 24040-32), and HyClone Cell Boost 5 supplement (Cytiva, Marlborough, MA, USA; Cat. No. SH3086501). CD BHK 21 production medium was supplemented with sodium bicarbonate (Sigma-Aldrich, St. Louis, MO, USA; Cat. No. S5761). Suspension cultures were maintained at 37 °C in a 5% CO_2_ atmosphere in a shaking incubator.

### 2.2. Viruses

Four different FMDV serotype O strains were used: two field isolates from outbreaks in the ROK, O/SKR/Jincheon/2014 and O/SKR/Boeun/2017, and two genetically engineered recombinant strains of foreign origin, O1 Manisa (R) and O PanAsia-2 (R), generated by replacing the P1 region of O1/Manisa/Turkey/69 and PanAsia-2, as previously reported [26,27,28,29] (Table 1).

### 2.3. Stabilizers

Sucrose (Sigma-Aldrich, St. Louis, MO, USA; Cat. No. S0389), glycerol (Sigma-Aldrich, St. Louis, MO, USA; Cat. No. S9012), and CVS, a proprietary thermostabilizing formulation developed and provided by CAVAC (Daejeon, Republic of Korea), were used in this study. The stabilizing composition (CVS) used in this study was provided by Choong Ang Vaccine Laboratories Co., Ltd. (CAVAC), the patent holder of KR102544928B1. According to the patent, CVS is composed of 13 L-amino acids, including L-methionine, L-valine, L-glutamine, L-asparagine, L-histidine, L-serine, L-alanine, L-glycine, L-threonine, L-proline, L-arginine, and L-aspartic acid at 2.5% (*w*/*v*) each, and L-glutamic acid at 5.0% (*w*/*v*). The formulation was prepared in distilled water, adjusted to pH 6.5–7.0, and filtered prior to use [30]. Sucrose was dissolved in distilled water to prepare a 60% (*w*/*v*) sucrose solution, whereas glycerol was diluted with distilled water to prepare a 50% glycerol solution.

Supernatants from BHK-21 suspension cultures infected with the four FMDV serotype O strains were mixed with 50% CVS (*v*/*v*) or with stabilizer formulations prepared by combining cell culture medium with the stabilizer stock solutions to obtain final concentrations of 3% sucrose or 10% glycerol.

### 2.4. Evaluation of the Thermostability of FMDV in the Presence of Different Stabilizers

Virus stocks of the four FMDV serotype O strains were prepared by mixing the virus with either cell culture medium or the stabilizer formulations to a final volume of 1 mL. To evaluate the FMDV thermostability, the virus–stabilizer mixtures were incubated at different temperatures and varying durations. These mixtures, containing virus with either stabilizers or cell culture medium, were stored at −80 °C, 4 °C, 25 °C, or 37 °C for 2, 4, or 7 d. Following storage under the indicated conditions, viral titers were determined using adherent BHK-21 cells and calculated according to the Reed–Muench method [31], and viral infectivity was expressed as the 50% tissue culture infectious dose (TCID_50_) per mL.

### 2.5. Effects of Stabilizers on Cell Viability

To evaluate the effects of stabilizers on BHK-21 suspension cell growth, cells were seeded at 3.0 × 10^5^ cells/mL in 30 mL of serum-free CD BHK-21 or ProVero-1 medium in shaking flasks. Following adaptation, the culture medium was replaced with fresh CD BHK-21 or ProVero-1 medium supplemented with either 3% (*w*/*v*) sucrose or 50% CVS (*v*/*v*) at the designated concentrations. Cells maintained in the corresponding medium without stabilizer supplementation served as the control group.

To assess cell growth and viability, cultures were incubated for 72 h, and 100 μL aliquots were collected at specific time points (0, 16, 24, 48, and 72 h). For cell counting, 10 μL of each cell suspension was mixed with an equal volume of trypan blue solution (Thermo Fisher Scientific, Waltham, MA, USA; Cat. No. 15250061), and 10 μL of the resulting mixture was loaded onto a TC20 counting slide (Bio-Rad, Hercules, CA, USA). Viable cell density and cell viability were determined using a TC20 Automated Cell Counter (Bio-Rad, Hercules, CA, USA).

### 2.6. Evaluation of Viral Growth

To analyze viral growth kinetics, BHK-21 suspension cells were seeded at a density of 3.0 × 10^5^ cells/mL in 30 mL of serum-free CD BHK-21 or ProVero-1 medium in shaking flasks. To evaluate the effects of stabilizers during infection, cells were inoculated with the four cell-adapted FMDV serotype O strains [O/SKR/Jincheon/2014, O/SKR/Boeun/2017, O1 Manisa (R), and O PanAsia-2 (R)] at a multiplicity of infection of 0.01 or 0.001. The cultures were maintained at 37 °C in a 5% CO_2_ shaking incubator. Supernatant samples were harvested at specific time points (0, 1, 8, 16, and 24 h post-infection), and viral titers were determined as the TCID_50_ using the method described above.

### 2.7. O/SKR/Jincheon/2014 Antigen Purification and Short-Term Stability Test

O/SKR/Jincheon/2014 antigen was purified as previously described [32]. Briefly, virus-containing supernatants were harvested from BHK-21 suspension cells following complete cytopathic effect (CPE) and clarified by centrifugation at 12,000 rpm for 20 min. The clarified supernatants were inactivated with 0.003 mM binary ethylenimine (BEI; Sigma-Aldrich, St. Louis, MO, USA; Cat. No. B65705) for 24 h and concentrated using 7.5% polyethylene glycol (PEG) 6000 (Sigma-Aldrich, St. Louis, MO, USA; Cat. No. 81253) and 2.3% NaCl (Sigma-Aldrich, St. Louis, MO, USA; Cat. No. S3014) with continuous stirring for 16 h at 4 °C. The concentrated material was resuspended in Tris-KCl buffer (50 mM Tris and 150 mM KCl, pH 7.4; Sigma-Aldrich, St. Louis, MO, USA; Cat. Nos. T1503 and P3911, respectively) and purified by ultracentrifugation through a 15–45% sucrose density gradient at 30,000 rpm for 4 h at 4 °C using an SW41Ti rotor (Beckman Coulter, Brea, CA, USA). The concentration of the purified 146S antigen was determined by measuring the absorbance at 259 nm using a spectrophotometer. Complete virus inactivation was confirmed by at least three serial passages in LF-BK and BHK-21 cells before the purified antigen was used in subsequent experiments.

For the short-term stability test, the purified O/SKR/Jincheon/2014 antigen was adjusted to a final concentration of 20 µg/mL of 146S antigen and formulated with the indicated stabilizers. The formulated antigens were stored in liquid form at 4 °C or 25 °C for up to 7 days. The 146S antigen content was determined at each designated time point by measuring absorbance at 259 nm. The percentage recovery of 146S antigen was calculated relative to the initial antigen content before storage using the following equation:

Recovery (%) = (146S antigen content after storage/146S antigen content before storage) × 100.

### 2.8. Statistical Analysis

**Figure 1:**
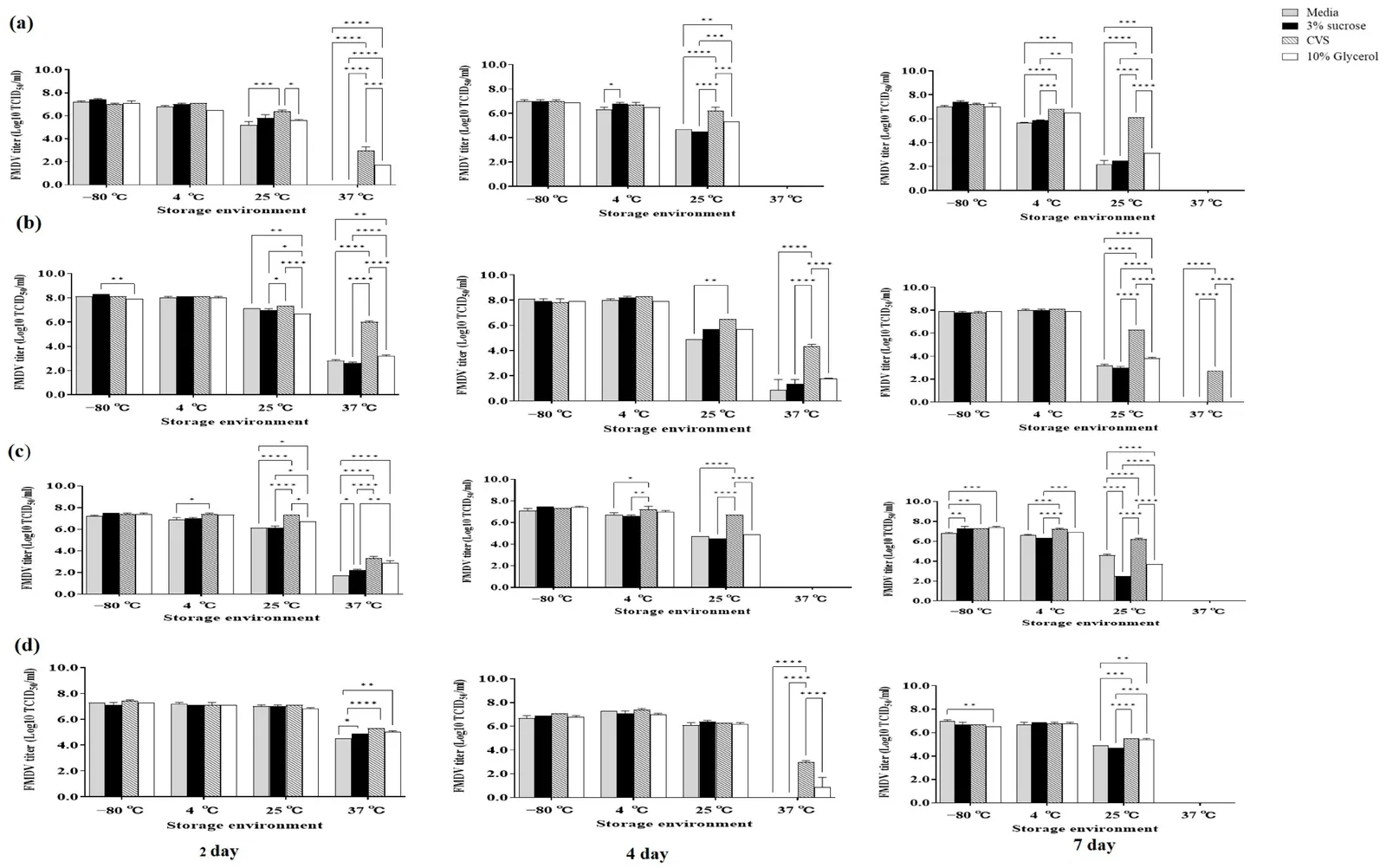
Evaluation of stabilizer efficacy under different temperatures and storage durations. Four FMDV serotype O strains, (**a**) O/SKR/Jincheon/2014, (**b**) O/SKR/Boeun/2017, (**c**) O1 Manisa (R), and (**d**) PanAsia-2 (R), were mixed with either stabilizers or cell culture medium (control) and stored at −80 °C, 4 °C, 25 °C, or 37 °C for 2, 4, or 7 d. Following storage under the indicated conditions, viral infectivity was determined by measuring the 50% tissue culture infectious dose (TCID_50_) in adherent BHK-21 cells. Data are presented as the mean ± standard deviation from three independent experiments (*n* = 3). Statistical significance was assessed using two-way ANOVA followed by Tukey’s multiple-comparisons test. Statistical significance was defined as * *p* < 0.05, ** *p* < 0.01, *** *p* < 0.001, and **** *p* < 0.0001.

**Figure 2:**
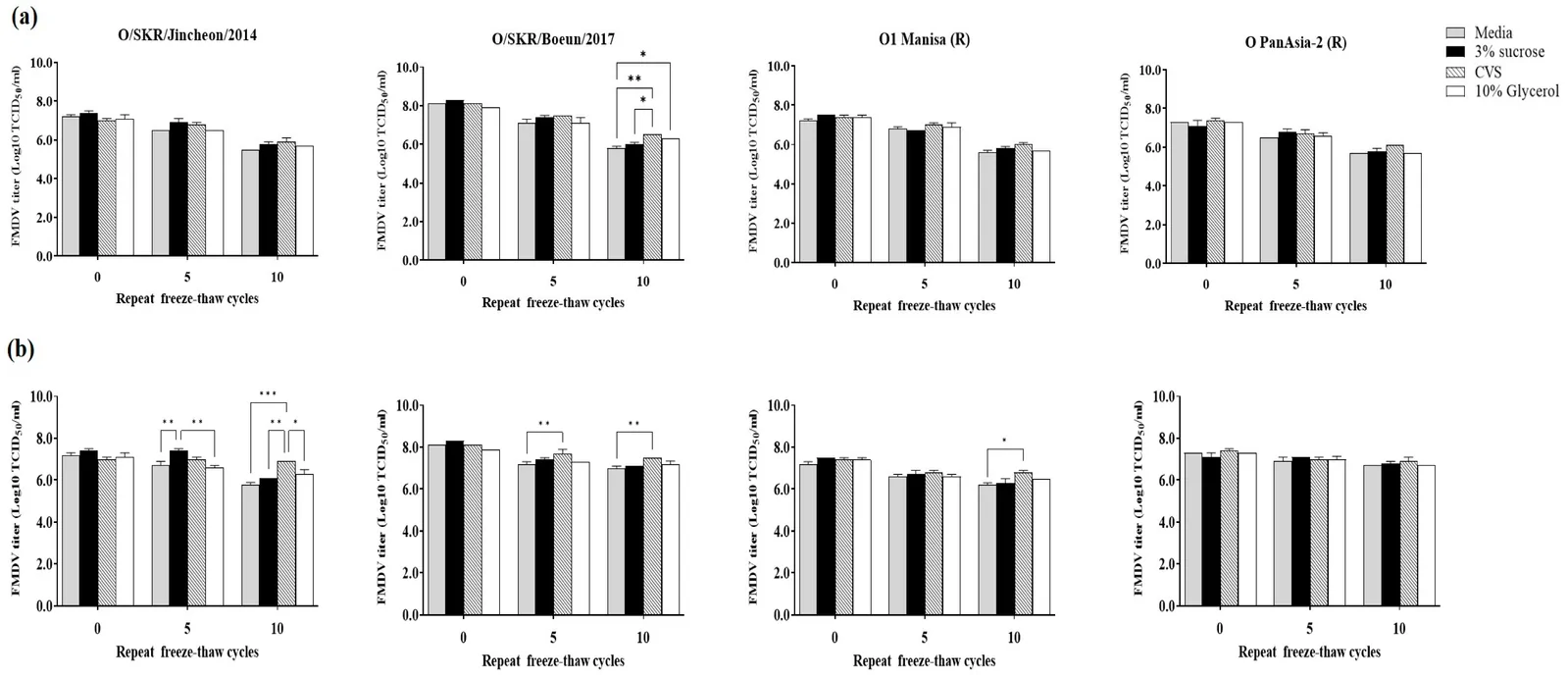
Evaluation of viral stability following repeated freeze–thaw cycles of FMDV formulated with different stabilizers after storage at −80 °C or in liquid nitrogen (LN_2_). Viral titers of four FMDV serotype O strains [O/SKR/Jincheon/2014, O/SKR/Boeun/2017, O1 Manisa (R), and O PanAsia-2 (R)] formulated with the indicated stabilizers were compared with those of the serum-free medium control following repeated freeze–thaw cycles between −80 °C or LN_2_ and 37 °C. Virus samples were stored either (**a**) at −80 °C or (**b**) in LN_2_ prior to freeze–thaw treatment. Viral infectivity was measured after 0, 5, and 10 freeze–thaw cycles. Data are presented as mean viral titers ± standard deviation. Statistical significance was determined by comparison with the serum-free medium control where applicable. Data are presented as the mean ± standard deviation from three independent experiments (*n* = 3). Statistical significance was assessed using two-way ANOVA followed by Tukey’s multiple-comparisons test. Statistical significance was defined as * *p* < 0.05, ** *p* < 0.01, and *** *p* < 0.001.

**Figure 3:**
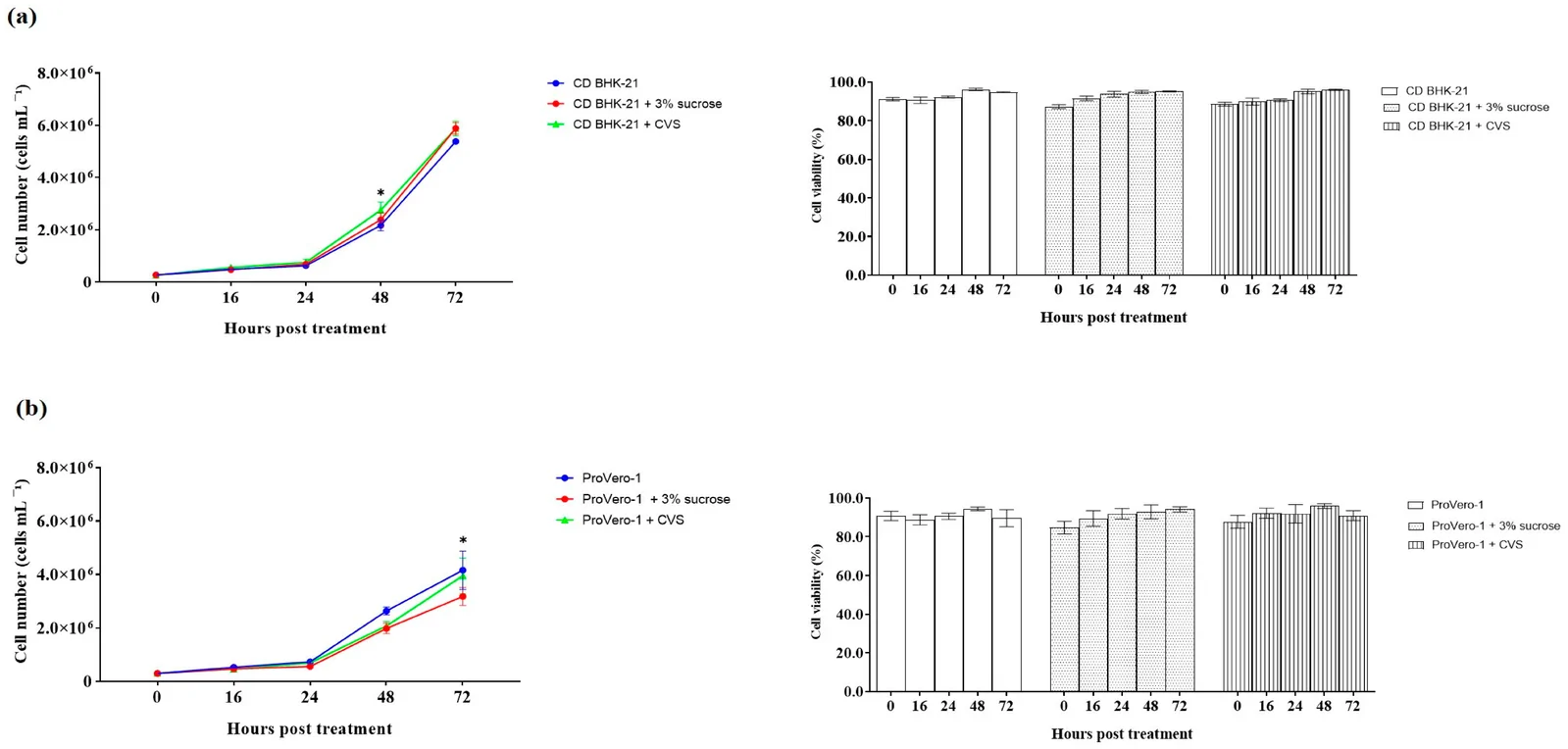
Effects of stabilizers on BHK-21 suspension cell growth and viability. BHK-21 suspension cells were cultured in CD BHK-21 or ProVero-1 medium and treated with either 3% sucrose or CVS. Cells were collected at specific time points (0, 16, 24, 48, and 72 h) after treatment to determine (**a**) total cell number and (**b**) cell viability. For cell number, statistical significance was assessed using two-way ANOVA followed by Tukey’s multiple-comparisons test, with treatment and time as the two factors. In contrast, cell viability (%) was presented for descriptive comparison of viability patterns, and no statistical significance testing was performed. Data are presented as the mean ± standard deviation (SD) of three independent experiments (*n* = 3), with triplicate measurements performed within each experiment. For cell number, statistical significance was defined as *p* < 0.05. * indicates *p* < 0.05.

**Figure 4:**
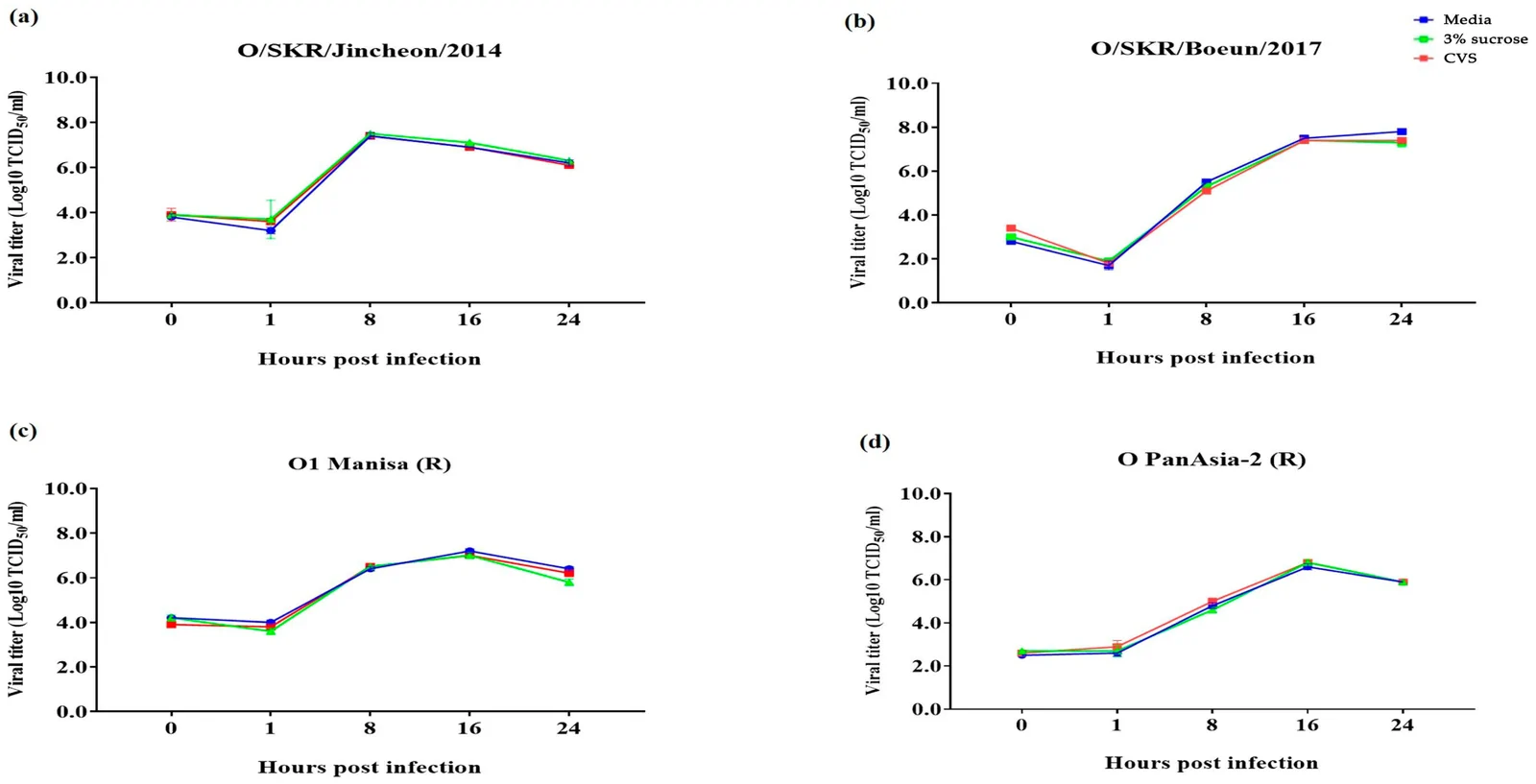
Effects of 3% sucrose and CVS on FMDV growth kinetics in BHK-21 suspension cells. Growth kinetics of four FMDV serotype O strains, (**a**) O/SKR/Jincheon/2014, (**b**) O/SKR/Boeun/2017, (**c**) O1 Manisa (R), and (**d**) O PanAsia-2 (R), were evaluated in BHK-21 suspension cells. Growth kinetics of viruses derived from 3% sucrose- or CVS-treated virus stocks were compared with those of unstabilized control virus stocks. Data are presented as the mean ± standard deviation of three independent experiments (*n* = 3), with triplicate measurements performed within each experiment. Statistical significance was assessed using two-way ANOVA followed by Tukey’s multiple-comparisons test. No statistically significant differences were observed among the stabilizer-treated and unstabilized control groups in viral growth kinetics.

Statistical analyses were performed using two-way analysis of variance (ANOVA) followed by Tukey’s multiple-comparisons test in GraphPad Prism version 9.0 (GraphPad Software, San Diego, CA, USA). All data represent the mean ± standard deviation (SD) of three independent experiments (*n* = 3), where each experiment’s value was calculated from technical triplicates. Two-way ANOVA with Tukey’s test was applied to assess significance across treatments and time points for thermostability, freeze–thaw, cell number, and viral growth kinetics data (Figure 1, Figure 2, Figure 3 and Figure 4). Cell viability (%) in Figure 3 was presented for descriptive comparison without formal hypothesis testing. Statistical significance is indicated as follows: * *p* < 0.05, ** *p* < 0.01, *** *p* < 0.001, and **** *p* < 0.0001.

## 3. Results

### 3.1. Thermostability of FMDV Strains Formulated with Different Stabilizers

The thermostability of four cell-adapted FMDV serotype O strains formulated with different stabilizers was evaluated by storing virus-containing culture supernatants at −80 °C, 4 °C, 25 °C, or 37 °C for up to 7 d (Figure 1).

Across the four FMDV serotype O strains, viral titers remained stable during storage at −80 °C and 4 °C throughout the 2-day storage period, regardless of the stabilizer used. At 25 °C, CVS provided greater maintenance of viral infectivity than the conventional stabilizers (3% sucrose and 10% glycerol) throughout the 7-day storage period. At 37 °C, CVS exhibited superior protective efficacy, maintaining detectable viral infectivity for up to 2 d across all four strains and for up to 4 d in O/SKR/Bouen/2017 and O PanAsia-2 (R). Notably, at this temperature, O/SKR/Bouen/2017 exhibited the greatest thermal stability among the four strains, maintaining detectable infectivity for up to 7 d.

The stabilizing effect of 10% glycerol on viral thermal stability was significantly greater than that of 3% sucrose after 7 d of storage at 25 °C, whereas 3% sucrose did not exhibit a significant stabilizing effect on viral thermal stability under the same conditions (Figure 1a–d).

### 3.2. Cryoprotective Efficacy of Different Stabilizers Against Freeze–Thaw Stress

The cryoprotective effects of the different stabilizers were evaluated by comparing viral titers following repeated freeze–thaw cycles after storage either at −80 °C or in liquid nitrogen (LN_2_), with serum-free medium serving as the unstabilized control (Figure 2).

During the first five freeze–thaw cycles following storage at −80 °C, no significant differences in viral titers were observed among the stabilizer formulations for any of the four FMDV serotype O strains. After 10 freeze–thaw cycles, only the O/SKR/Boeun/2017 formulated with CVS maintained significantly higher viral infectivity than both the unstabilized control and the 3% sucrose formulation. In contrast, no significant stabilizer-dependent protective effects were observed for the other strains under the same conditions (Figure 2a).

Following storage in LN_2_, a similar overall pattern was observed, although the protective effect of CVS was more pronounced. For the O/SKR/Jincheon/2014 strain, CVS-treated virus maintained significantly higher infectivity than the unstabilized control and viruses treated with other stabilizer formulations, even after 10 freeze–thaw cycles. Similarly, for the O/SKR/Boeun/2017 and O1 Manisa (R) strains, CVS-treated virus exhibited significantly higher infectivity than the unstabilized control. In contrast, for the O PanAsia-2 (R) strain, CVS treatment did not confer a significant protective effect against LN_2_ storage and repeated freeze–thaw conditions (Figure 2b).

### 3.3. Effects of Stabilizers on BHK-21 Suspension Cell Growth

The effects of the viral stabilizers on host cell growth were evaluated using BHK-21 suspension cells cultured in the presence of virus preparations formulated with either 3% sucrose or CVS. Cell growth was compared with that of control cultures maintained in the corresponding culture medium (CD BHK-21 or ProVero-1) without stabilizer supplementation.

To evaluate the potential effects of the stabilizers on BHK-21 suspension cell growth, cell number and viability were monitored over 72 h in CD BHK-21 and ProVero-1 media. Cell numbers increased progressively over time in all treatment groups in both media. In CD BHK-21 medium, the CVS-treated group showed a significantly higher cell number than the untreated control at 48 h (*p* < 0.05). Similarly, in ProVero-1 medium, cell numbers increased throughout the observation period, with CVS-treated cells showing a growth pattern comparable to that of the control, whereas the 3% sucrose-treated group showed a lower cell number at 72 h (*p* < 0.05). Cell viability remained generally high throughout the 72 h observation period in both media, with no apparent reduction following treatment with either 3% sucrose or CVS (Figure 3a,b).

Nevertheless, because FMDV is harvested within 24 h post-infection, it was confirmed that there was no effect of stabilizers on cell growth.

### 3.4. Effects of CVS on FMDV Growth in BHK-21 Suspension Cells

The effects of CVS on viral growth were evaluated in BHK-21 suspension cells using four FMDV serotype O strains [O/SKR/Jincheon/2014, O/SKR/Boeun/2017, O1 Manisa (R), and O PanAsia-2 (R)]. Viral titers were compared among 3% sucrose- or CVS-treated viruses and the unstabilized control. No significant differences in viral growth were observed between either stabilizer-treated group and the unstabilized control for all four FMDV strains (Figure 4a–d).

## 4. Discussion

The preservation of FMDV is critical for maintaining seed virus quality throughout vaccine development, production, and long-term storage. Because FMDV is highly susceptible to thermal and physical degradation, loss of viral infectivity during storage can directly affect vaccine quality and manufacturing consistency [19,33,34]. Therefore, the development of effective stabilizers that preserve viral infectivity while maintaining viral characteristics is essential for reliable seed virus management [35]. In the ROK, approximately USD 53 million is spent annually on FMD vaccination programs, and substantial efforts are being directed toward establishing domestic FMD vaccine production [8,10,36]. A key prerequisite for successful localization of vaccine production is the establishment and long-term preservation of high-quality seed viruses [37].

Among the seven FMDV serotypes, serotype O is considered one of the least structurally stable, and certain strains, including O/SKR/Jincheon/2014, exhibit pronounced capsid instability. This intrinsic instability can result in the rapid dissociation of intact 146S particles into 12S subunits during antigen production and storage, thereby reducing the preservation of antigenic mass and posing a significant challenge to maintaining vaccine potency during extended storage [19,38,39]. Moreover, regeneration of seed viruses through repeated propagation inevitably increases passage numbers, which may alter viral growth characteristics, antigenicity, and genetic stability, thereby complicating long-term seed virus management [40,41]. Therefore, selecting an effective stabilizer capable of minimizing loss of viral infectivity during storage is of considerable practical importance [42].

Previous studies have demonstrated that sucrose, glycerol, proteins, amino acids, and other polyol-based additives can enhance FMDV stability by protecting viral capsids against thermal denaturation and structural disruption during storage [24,43,44]. Based on these findings, the present study compared two conventional stabilizers (3% sucrose and 10% glycerol) with the newly formulated CVS stabilizer using four cell-adapted FMDV serotype O strains, including both field isolates and recombinant vaccine strains. CVS provided superior protection under conditions of significant environmental stress. Although all stabilizer formulations maintained viral infectivity during storage at −80 °C and 4 °C, differences among the formulations became apparent with increasing storage temperature (Figure 1). In particular, CVS consistently preserved significantly higher viral titers at 25 °C and 37 °C than either 3% sucrose or 10% glycerol, with its protective effect becoming increasingly evident with prolonged storage. Interestingly, O/SKR/Boeun/2017 exhibited relatively greater intrinsic thermal stability than the other strains in the presence of the stabilizer, suggesting that the stabilizing efficacy may, at least in part, depend on the inherent physicochemical properties of individual virus strains.

To further investigate the structural basis of this stabilizing effect, the 146S antigen stability of O/SKR/Jincheon/2014, which exhibits pronounced capsid instability, was evaluated. Despite its pronounced instability, CVS effectively maintained 146S antigen stability for up to 4 days at 4 °C and 25 °C, outperforming 10% glycerol particularly at 25 °C (Figure S1). These findings suggest that CVS may also have potential for stabilizing FMDV vaccine antigens by preserving the structural integrity of 146S particles. The stabilizing effect of the amino-acid-based CVS formulation may involve preferential hydration/exclusion effects of amino acids and modulation of protein–solvent interactions, thereby reducing conformational destabilization and aggregation of viral structural proteins [19,45,46]. However, whether CVS can maintain 146S stability during vaccine formulation and in the final vaccine product remains to be experimentally determined, and the precise molecular mechanism underlying its stabilizing effect requires further investigation.

Repeated freeze–thaw cycles represent another important cause of infectivity loss during long-term storage, as ice crystal formation and osmotic stress can damage viral particles [37,47,48]. In the present study, all formulations provided adequate protection of the viruses during the initial freeze–thaw cycles. However, particularly for O/SKR/Jincheon/2014, viruses treated with CVS following storage in LN_2_ consistently exhibited higher viral infectivity than those treated with 3% sucrose or 10% glycerol after ten freeze–thaw cycles (Figure 2b). In terms of thermal stability, the effects of CVS treatment varied among the strains. This finding further supports the possibility that intrinsic differences in capsid stability among FMDV strains contribute to differences in their resistance to thermal stress. Therefore, the effectiveness of a particular stabilizer may depend on the structural characteristics and inherent stability of the viral capsid [48,49].

An effective virus stabilizer should preserve viral infectivity without affecting virus production. Importantly, neither 3% sucrose nor CVS affected the growth of BHK-21 suspension cells compared with the corresponding culture medium controls (Figure 3). In addition, the viral growth kinetics of all four serotype O strains were comparable among CVS-treated, sucrose-treated, and untreated virus preparations, indicating that CVS does not impair viral replication (Figure 4). Together, these findings demonstrate that the enhanced virus stability conferred by CVS is achieved without reducing cell viability or virus productivity, supporting its compatibility with existing FMD vaccine manufacturing processes. Given the widespread use of BHK-21 suspension cells in large-scale commercial vaccine production, this characteristic represents an important advantage for industrial application [50,51]. The potential applicability of CVS beyond FMDV vaccines is supported by its use in the development of a commercial rabies bait vaccine [52]. Although FMDV vaccine stability is commonly assessed based on the preservation of intact 146S particles, stabilization of rabies and other viral vaccines requires maintenance of viral particle integrity, infectivity, and antigenicity. Thus, CVS may have broader potential as an amino acid-based stabilizer for viral vaccines. However, its efficacy and underlying mechanisms should be experimentally validated for each virus, particularly for structurally distinct viruses such as PPRV and LSDV.

Collectively, the present study demonstrates that CVS effectively enhances both thermal stability and cryoprotection of FMDVs while maintaining normal cell growth and viral replication. Improved preservation of seed viruses could reduce infectivity loss during storage and minimize the need for repeated virus propagation, thereby potentially reducing the risk of passage-associated changes by increased passaging in viral characteristics. Such improvements could facilitate more reliable long-term management of vaccine seed viruses and contribute to the stable production of domestically manufactured FMD vaccines.

This study has several limitations. First, only FMDV serotype O strains were evaluated, and viral stability was assessed primarily using infectivity assays. Serotype O was selected because it is the predominant vaccine serotype used in the ROK and is generally considered less stable compared with other FMDV serotypes. Nevertheless, the inclusion of both field isolates and recombinant vaccine strains contributed to the practical relevance of the study. Further studies are warranted to determine whether CVS provides comparable stabilizing effects against other FMDV serotypes, including serotypes A, Asia1, and SAT1, and whether it preserves the structural integrity of the 146S antigen, the principal immunogenic component of inactivated FMD vaccines [53,54,55]. Additionally, further studies, including long-term storage studies, scale-up manufacturing evaluations, and vaccine efficacy assessments in target animals, will be required to establish the practical utility of CVS [54,56]. Overall, the present findings suggest CVS as a promising stabilizer for FMDV seed virus preservation and its potential applications in stabilizing other FMDV serotypes and temperature-sensitive veterinary viruses.

## 5. Conclusions

This study demonstrates that the novel stabilizer CVS significantly improves the thermo- and cryostability of cell-adapted FMDV serotype O strains, including both domestic field isolates and recombinant strains. This finding is noteworthy because FMDV serotype O is known to be relatively less thermostable than other serotypes. Compared with the conventional stabilizers, CVS improved thermo- and cryostability without adversely affecting cell growth or viral replication. However, the stabilizing effect varied among strains, suggesting a strain-dependent response. Overall, CVS has potential as an effective stabilizer for the preservation and vaccine manufacturing of FMDV serotype O, although further studies are needed to validate its applicability to other serotypes and viruses.

## Figures and Tables

**Table 1:** List of FMDV serotype O strains used in this study.

Original	FMDV	Topytype /Genotype	Virus Adapted Media	Passage	Reference
Field isolates (Outbreaks in the ROK)	O/SKR/Jincheon/2014	SEA/Mya-98	CD BHK-21	L5B5SB3	[26]
	O/SKR/Boeun/2017	ME-SA/Ind-2001	CD BHK-21	L5B4SB8	[27]
Recombinant (P1 sequence)	O1 Manisa (R)	ME-SA	CD BHK-21	Z5B8SB4	[28]
	O PanAsia-2 (R)	ME-SA/PanAsia-2	ProVero-1	Z5B5SB5	[29]

Z, adherent ZZ-R 127; L, adherent LF-BK cell; B, adherent BHK-21 cell; SB, suspension BHK-21 cell.

## Data Availability

The original contributions presented in this study are included in the article/Supplementary Material. Further inquiries can be directed to the corresponding authors.

## Supplementary Materials

The following supporting information can be downloaded at: https://www.mdpi.com/article/10.3390/microorganisms14092051/s1. Figure S1: Short-term stability of O/SKR/Jincheon/2014 antigens formulated with different stabilizers.

## Abbreviations

The following abbreviations are used in this manuscript:

FMD	foot-and-mouth disease
FMDV	foot-and-mouth disease virus
ROK	Republic of Korea
ZZ-R 127	fetal goat tongue tissue cells
BHK-21	baby hamster kidney cells
LF-BK	fetal porcine kidney cells
TCID_50_	50% tissue culture infectious dose
LN_2_	liquid nitrogen
CAVAC	Choong Ang Vaccine Laboratories Co., Ltd.
CPE	complete cytopathic effect
ANOVA	analysis of variance
