# Supplementary material for: Novel Thermostabilizing Formulation for Enhanced Stability of Foot-and-Mouth Disease Virus During Storage and Environmental Stress
Source: Microorganisms. 2026 Sep 14;14(9):2051. doi: 10.3390/microorganisms14092051 (PMC13609129; doi:10.3390/microorganisms14092051)
Supplement: Supplementary file 1 [file microorganisms-14-02051-s001.zip › microorganisms-4541972-supplementary.pdf]

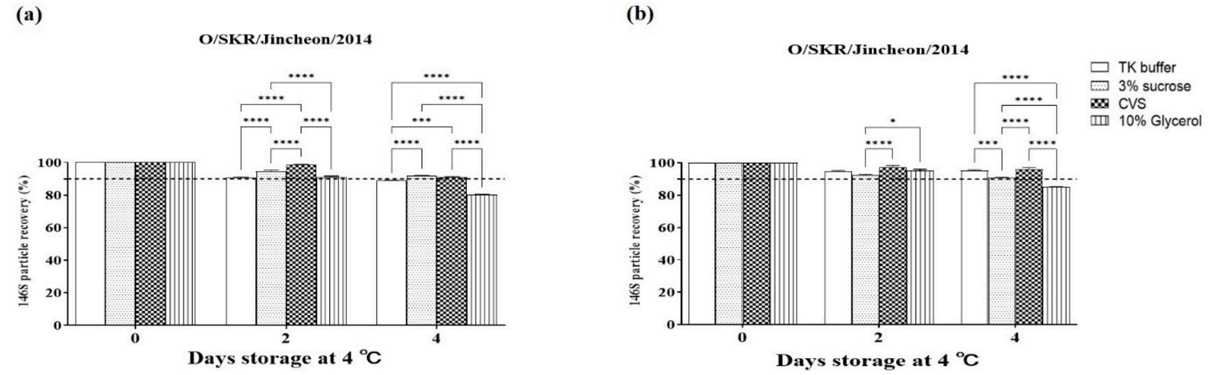


**Figure S1.** Short-term stability of O/SKR/Jincheon/2014 antigens formulated with different stabilizers. O/SKR/Jincheon/2014 antigen containing 20 μg/mL of 146S antigen was formulated with the indicated stabilizers and stored in liquid form at 4 °C or 25 °C for up to 4 days. The 146S antigen concentration was determined by measuring absorbance at 259 nm using a spectrophotometer. The recovery rate of 146S antigen was calculated at each time point by comparing the antigen content after storage with that measured before storage, according to the following equation:

Recovery (%) = (146S antigen content after storage / 146S antigen content before storage) × 100.

Data are presented as the mean ± standard deviation (SD) of three independent experiments (n = 3). Statistical significance was assessed using two-way analysis of variance (ANOVA) followed by Tukey's multiple-comparisons test. The dotted line indicates 90% antigen recovery. * $p < 0.05$, ** $p < 0.01$; *** $p < 0.001$, and **** $p < 0.0001$.
